# Empathizing with sensory and movement differences: moving toward sensitive understanding of autism

**DOI:** 10.3389/fnint.2013.00038

**Published:** 2013-05-24

**Authors:** Steven K. Kapp

**Affiliations:** Human Development and Psychology Division, Graduate School of Education and Information Studies, University of California Los AngelesLos Angeles, CA, USA

The autism diagnosis requires deficits in social interaction and communication, yet neither occurs in isolation. This brief literature-based analysis provides evidence that other factors are involved in autistic people's atypical social communication. The brain is a complicated system where regions serve multiple, general, and overlapping roles. Sensorimotor and broad cognitive processes underlie both neurotypicals' and autistics' social cognition and behavior. Sensory strengths sometimes underlie autistic people's difficulties, especially in dynamic contexts that require multimodal integration. Social abilities and behaviors occur between people in social contexts, and autistic and neurotypical people share mutual difficulties in understanding one another. This paper challenges attempts to reduce autism to social deficits, and suggests the need for better interpersonal and societal understanding of and support for autistic people.

## Integrative neuroscience

Increasing evidence supports how brain networks integrate complex information, including the contribution of sensorimotor areas to abilities and behaviors considered social in autistic and neurotypical people. A recent study that sought to identify the components of autistic people's “social brain” identified a sensorimotor circuit as one of the subsystems (Gotts et al., 2012). Typically, as people learn and make sense of things, different parts of the brain are well-connected and function in sync and rhythm with one another, with activity oscillating back and forth (Wang, 2010; Uhlhass and Singer, 2012). Such wiring contributes to the rhythm and synchrony of typical social interaction, but these processes happen atypically in autistic people (for example, greater or less connectivity in certain areas compared with neurotypicals; Mostofsky and Ewen, 2011; Gomot and Wicker, 2012; Uddin et al., in press). Similarly, the cerebellum (Fatemi et al., 2012), basal ganglia (Qiu et al., 2010; Prat and Stocco, 2012), and sensorimotor cortex (Hamilton, 2013) brain structures known to assist motor control also connect to other regions and appear to play important roles in timing, speech production (Bouchard et al., 2013), the back-and-forth conversation (Scott et al., 2009) that is often problematic for autistic people.

A brain region called the insula exemplifies the complexity of challenges facing autistic people. Once considered to play a limited and isolated role (its name means “island”; Craig, 2010), the insula connects to diverse brain regions (Kurth et al., 2010; Deen et al., 2011). It is a key part of a brain network that integrates external sensory stimuli with one's own bodily, emotional, and mental states (Uddin and Menon, 2009), and which may best distinguish autism (Uddin et al., in press). Regarding the insula's role in subserving interoception (awareness of internal bodily stimuli; Craig, 2009), many autistic people are hypersensitive to pain (Nader et al., 2004) and can even have a highly accurate sense of their own heartbeat (Cascio et al., 2013). Interoception and the insula also contribute to a variety of social functions (Di Martino et al., 2009; Herbert and Pollatos, 2012), such as sharing attention with others (Mundy et al., 2010), and awareness of (Silani et al., 2008; Bird et al., 2010; Herbert et al., 2011) and verbal expression (Saxbe et al., 2012) of one's own emotions. Most autistic people have difficulties with interpreting and expressing their own emotions, but those more able to do so are less likely to have challenges with recognizing others' emotions (Bird et al., 2010), interpreting their facial expressions (Cook et al., 2013), or with making eye contact (Bird et al., 2011).

The insula also plays a role in unpleasant situations (Wicker et al., 2003; Wright et al., 2004; Jabbi et al., 2008). It contributes to autistic people's tendency for hypersensitivity to unpleasant textures, which—alongside hyposensitivity to other textures (Foss-Feig et al., 2012)—relates mostly to social impairment (Cascio et al., 2012b). Moreover, the insula is involved in the processing of norm violations (Sanfey et al., 2003), and autistic people show enhanced activation of the insula when rules are broken, which can create a false appearance (including in the insula) of reduced concern about social exclusion (Bolling et al., 2011; Masten et al., 2011). Indeed, the insula is involved in cognitive flexibility, including attention switching (Menon and Uddin, 2010) and tolerance for uncertainty, as well as understanding others' emotions (Singer et al., 2009). These are related, because people cannot mind-read, but rather approximate others' emotions and thoughts through probabilistic inference based on experience (Gopnik, 2011; Gopnik and Wellman, 2012).

## Mind-body interaction

Rather than relying on discrete social domains, interpreting other people's thoughts and emotions from their behavior or communication requires more general processes (Gernsbacher and Frymiare, 2005; Wilkinson and Ball, 2012). Typically, reading nonverbal cues involves sensorimotor and basic attentional processes, and happens relativelyautomatically and unconsciously (Pineda and Hecht, 2009; Frith and Frith, 2012). Autistic people tend to have significant challenges with all these abilities (Ben-Sasson et al., 2009; Kapp et al., 2011; Donnellan et al., 2013). Such challenges with reading body language relate to general trouble with movement; slowing down nonverbal cues significantly improves accuracy of processing them (Gepner and Féron, 2009). Autistics often demonstrate competence when processing the same stimulus when static but difficulties when in motion (Hanley et al., 2012; Weisberg et al., 2012). For example, many autistic people have oculomotor control (eye movement) problems, which challenge joint attention and language development (Mundy et al., 2009; Gliga et al., 2012; Kelly et al., 2013). In particular, many autistic people's pupils reflect intense activity in the nervous system, which challenges quick, coordinated, spontaneous attention (Anderson et al., 2012). Faced with these difficulties, most autistic people learn to rely on more advanced active reasoning skills to infer body language (Ahmed and Miller, 2011; Vivanti et al., 2011; Senju, 2013).

Like sensory processing (Aglioti and Pazzaglia, 2011) and movement (Riley et al., 2012) in neurotypicals, sensorimotor differences in autistic people underlie various behaviors impacting social functioning. For example, sensory hypersensitivity and integration difficulties often lead to social withdrawal from overload (Reynolds et al., 2011; Brock et al., 2012), while the slow responsiveness from sensory hyposensitivity distinctively contributes to autism-related impairment (Ben-Sasson et al., 2009; Brock et al., 2012). Furthermore, challenges with body posture and gestures, listed as impairment in social interaction in the autism diagnosis (APA, 2000), relate to respective difficulties with postural control (from poor balance; Travers et al., 2012) and performing skilled movements (related to dyspraxia: impairment in motor planning; Dziuk et al., 2007). Similarly, atypical social distance (personal space; Frazier et al., 2012) may stem from problems sensing and orienting to one's body in space (Blanche et al., 2012).

Moreover, as in the general population (Niedenthal, 2007; Barsalou, 2008), emotions and language in autistic people are grounded in the body. When autistics have challenges with social emotions, these draw from embodied emotion dysregulation more broadly (Winkielman et al., 2009; Mazefsky et al., 2012). Likewise, when autistics have challenges understanding figurative language and other aspects of what often gets labeled as pragmatics (language applied to social contexts), this stems from general challenges with receptive (understanding) language (Gernsbacher and Pripas-Kapit, 2012). While language is acquired through social contexts, speech requires the coordination of many muscles; most autistics have atypical speech (whether functional or not), including unusual prosody (rate, rhythm, volume, pitch, and tone; Eigsti et al., 2011).

Although proponents of social deficit theories of autism often emphasize poor autobiographical memory, this originates in part from the sense of smell and broader memory problems. Certain odors automatically induce memories and social contact in neurotypicals (Larsson and Willander, 2009), but the effect may tend to be limited to more familiar events and people in autistics (Parma et al., 2013). Indeed, a few studies have linked taste-smell processing difficulties in autistics with greater communication and behavioral challenges (Hilton et al., 2010; Lane et al., 2011). Moreover, autistics tend to have challenges with not only past- but also future-oriented memory (prospective memory: remembering to carry out intentions); this contributes to planning, organization, multitasking, and social cognitive challenges (Rajendran et al., 2011; Lind and Williams, 2012; Williams et al., 2012).

## Complex differences

Any comprehensive theory of autism requires recognizing the complex nature of differences, including strengths and impairments that sometimes arise from them, as illustrated in the visual and auditory modalities. Visual strengths relate positively to language and other communication challenges (Atkinson, 2009; Joseph et al., 2009; Hubbard et al., 2012; Ohta et al., 2012); most autistics considered “untestable” can demonstrate visuospatial skills (Courchesne et al., 2012). Autistics tend to have enhanced ability, and natural orientation, to directly process visual stimuli (Happé and Frith, 2006; Mottron et al., 2006; Simmons et al., 2009), including the abilities to search for objects amid distractors, see patterns, and notice subtle changes in scenery (Simmons et al., 2009). Yet, for some, this hypersensitivity means pain (Kleinhans et al., 2010) or distraction (Doherty-Sneddon et al., 2012) from eye contract or bright lights (Fan et al., 2009), and aversion to change related to heightened recognition of subtle changes in the environment (Cléry et al., 2013a, b).

Similarly, autistic people's auditory strengths relate positively to their language challenges (Bonnel et al., 2010). Autistics tend to have greater perception of singular auditory stimuli such as absolute (“perfect”) pitch (Happé and Frith, 2006; Mottron et al., 2006; O'Connor, 2012), but hypersensitivity can mean greater pain from loud noise (Egelhoff and Lane, 2013), impairment in filtering out background noise (Lane et al., 2010; Egelhoff and Lane, 2013), and difficulty learning spoken words (Norbury et al., 2010). Because of general challenges with audiovisual integration when watching and listening to speech (Woynaroski et al., 2013), autistics tend to look at the mouth, which provides audiovisual synchrony (lip motion with speech sound; Klin et al., 2009) that helps autistics and typically developing infants develop language skills (Norbury et al., 2009; Young et al., 2009; Falck-Ytter et al., 2010; Lewkowicz and Hansen-Tift, 2012).

Indeed, the greatest differences often stem from simultaneous multisensory processing and integration of information more broadly. For example, related to visual-motor integration challenges, many autistics learn new movements (Haswell et al., 2009; Izawa et al., 2012) and facial expressions (Wright et al., 2008) by focusing on feedback from the body more than visual observation; autistics with especially low body awareness may struggle greatly with motor skills and communication (Freitag et al., 2007; Blanche et al., 2012; Linkenauger et al., 2012). Neurotypicals unconsciously integrate information, and their prior experiences and expectations shape their perception of surroundings (Schroeder et al., 2010) Autistics are also affected by this phenomenon, but more independent processing grounded in details of the environment can translate to more realistic perception (Brock, 2012; Cascio et al., 2012a; Pellicano and Burr, 2012). Yet for many this also means overwhelm and confusion in everyday settings that require dynamic online (in the moment) integration (Dinstein et al., 2012), and lack of automatic attention (but generally not inability to understand) the “big picture” or context, which contributes to communication challenges (Happé and Frith, 2006).

## Person-environment (social) interaction

Despite their inclusion in the autism diagnosis as an internal problem, communication, reciprocity, and relationships happen between people and must happen both ways to function (Donnellan et al., 2013). According to the concept of synchrony, effective communication happens not only between regions of a person's brain, but between communication partners, whose brains and bodies in turn will typically reflect mutual engagement (Hari et al., 2013). While people typically show neural synchrony when engaged in joint activity, autistic people and neurotypical communication partners both have challenges connecting with one another, demonstrated neurologically and behaviorally (Tanabe et al., 2012; Schilbach et al., in press). In spite of the listing of impairment in peer relationships within the autism diagnosis (APA, 2000), peers regularly bully and reject autistics, and are generally more likely to do so if the autistic person gets upset (Rieffe et al., 2012) or withdraws (Humphrey and Symes, 2010). Such stressful experiences cause and exacerbate co-occurring mental and physical conditions (Kohane et al., 2012), and present greater challenges for coping with autism.

Supporting autistic people requires flexibility between autistics and communication partners (Muskett et al., 2010). For example, autistic children tend to build more skills when their parents understand and accept them (Kapp et al., 2013; Oppenheim and Koren-Karie, unpublished). Such sensitivity requires learning why someone has particular behavior and working with the person (Amos, 2013); even challenging behavior may represent an adaptive form of compensatory communication (Damico and Nelson, 2005). Parents who understand the reasons for their autistic children's behaviors and learn to speak their child's language help their child gain skills in the parent's language, especially for more language delayed or impaired children, by becoming in sync with their child (Kasari et al., 2008; Perryman et al., 2012; Haebig et al., 2013; Siller et al., 2013).

Now that the autism field has begun to intensively study sensory-movement differences, they have become better understood, with potential to spur change. Autistics' challenges with sensory processing, motor skills, emotion regulation, and executive functioning often mask the extent or expression of their social understanding or interest in neurotypical contexts. Neurotypicals do not naturally recognize the full reasons for sensory-movement differences, and their centrality to communication differences, because they involve areas they process intuitively. Critically, as scientific evidence on the presence and importance of autistic people's sensory-movement differences mounts, it increasingly reflects autistic people's lived experiences (Chamak et al., 2008; Davidson and Henderson, 2010; Robledo et al., 2012). What society does with this knowledge will test everyone's sensitivity and understanding.
